# Dynamic sex-specific responses to synthetic songs in a duetting suboscine passerine

**DOI:** 10.1371/journal.pone.0202353

**Published:** 2018-08-29

**Authors:** Adam R. Fishbein, Julia Löschner, Julie M. Mallon, Gerald S. Wilkinson

**Affiliations:** 1 Program in Neuroscience and Cognitive Science, University of Maryland, College Park, Maryland, United States of America; 2 Department of Psychology, University of Maryland, College Park, Maryland, United States of America; 3 Animal Physiology, Institute for Neurobiology, University of Tübingen, Tübingen, Germany; 4 Department of Biology, University of Maryland, College Park, Maryland, United States of America; Texas Christian University, UNITED STATES

## Abstract

Many bird species produce temporally coordinated duets and choruses, requiring the rapid integration of auditory perception and motor production. While males and females of some species are known to participate in these displays for sex-specific purposes, few studies have identified perceptual features that trigger sex-specific contributions of coordinated song. Especially little is known about perception and production in duetting suboscine passerines, which are thought to have innate songs and largely static, rather than dynamic, vocal behavior. Here, we used synthetic stimuli in a playback experiment on chestnut-backed antbirds (*Myrmeciza exsul)* to (1) test whether differences in song frequency (Hz) can trigger sex-specific vocal behavior in a suboscine passerine (2) test for the functions of duetting in males and females of this species, and (3) determine whether these suboscines can dynamically adjust the temporal and spectral features of their songs. We found sex-specific responses to synthetic playback manipulated in song frequency (Hz), providing evidence that in this context males sing in duets for general territory defense and females join in for mate guarding purposes. In addition, we found that the birds altered the frequency, duration, and timing of their songs depending on the frequency of the playback songs. Thus, we show that these birds integrate spectral and temporal information about conspecific songs and actively modulate their responses in sex-specific ways.

## Introduction

Sex differences in vocal behavior are widespread among birds [1–4]. For species where only males sing, these differences are theorized to have evolved through female choice and male-male competition [5–7]. For species where females sing, the trait is thought to have evolved largely through social selection [8, 9]. For tropical species that hold territories year-round, males and females also often sing together in temporally coordinated duets [10, 11]. These joint displays often contain sex-specific notes and can facilitate cooperation, benefitting both partners (e.g. joint territory defense), conflict between the partners (e.g. mate guarding), or both purposes depending on the context, species, and season [9, 12, 13].

Sex-specific vocal behavior can also indicate the functions of duetting, a topic that has been studied more in oscine passerines, a suborder in which birds learn their songs, than suboscines, a suborder for which all species are thought to lack vocal learning ability [14–17] (but see [18, 19]). A number of non-mutually exclusive hypotheses have been proposed for the functions of duetting, and in most well-studied species duets have been found to have multiple functions [13, 20]. The most well supported cooperative function of duetting is joint resource defense, where the duet signals the presence of birds ready to defend the territory [20]. If duets serve the function of joint-territory defense, then males and females are expected to respond to intruders together rather than independently because duets are expected to be more threatening than solo songs [13]. Functions for duetting based on conflict between the partners have been reported less commonly, with the most well supported being mate-guarding [20]. Under this hypothesis, birds should perceive same-sex intruders as more threatening than intruders of the opposite sex and solitary intruders as more threatening than paired intruders [13].

Communally signaling birds–those that produce joint acoustic displays between two (duets) or more (choruses) individuals [21]–also provide excellent model systems for investigating the links between auditory perception and vocal production, as the animals need to integrate acoustic input with motor commands on a short time scale, making “on the spot adjustments” to create coordinated songs [13, 22]. While communally signaling animals must perceptually track conspecifics in order to vocalize at the appropriate time, few studies have attempted to identify the acoustic cues, such as song frequency, used by signalers in this process [23] (but see [24]). Sex differences in cues used for communal signaling must be common as sex-specific contributions to duets are widespread [20]; however, this question has rarely been addressed using controlled experiments.

In addition, little is understood about control of vocal production in duetting and chorusing suboscines [25, 26]. In oscines, active control of the syringeal muscles allows birds to finely manipulate the timing and fundamental frequency of their vocalizations [27, 28]. Because suboscines lack the forebrain motor circuitry present in oscines for controlling the syringeal muscles, it is widely assumed that they have little flexibility to adjust the acoustic features of their innate songs [29]. But evidence from communally signaling birds show that some suboscines can adjust the timing of their songs [30, 31]. Several suboscines have also been shown to modify the frequency of their songs to avoid anthropogenic or biological noise, but overall songbirds are able to adjust their vocalizations better to noisy environments [32, 33]. While Amador et al. [34] showed that song frequency in suboscines can be altered by changes in air sac pressure, current evidence suggests that spectral modification is rare among suboscines.

To examine sex differences in the dynamic adjustment of song in a suboscine, we studied the vocal responses of chestnut-backed antbirds (*Myrmeciza exsul)* to synthetic male solo, female solo, and duet song playback. This species is highly sedentary, sexually dichromatic, socially monogamous, and forms pairs that hold territories year-round [35, 36]. In this study, we had three objectives: (1) test whether differences in song frequency (Hz) can trigger sex-specific vocal behavior in this species; (2) determine whether there are sex differences in the form and function of duetting in this species through measuring vocal and movement behavior of the birds; and (3) test whether chestnut-backed antbirds can dynamically adjust the temporal and spectral features of their songs. We predict that (1) differences in frequency will trigger sex-specific vocal behavior, serving as a cue for sex identification in this species; (2) duets in chestnut-backed antbirds will function for joint-territory defense, as it is the most well-supported function for duetting across species [20] and (3) the birds will dynamically adjust the timing, but not the frequency, of their songs.

## Methods

### Subjects and study site

The chestnut-backed antbird is a common medium-sized (28g) passerine found near the ground in lowland neotropical rainforest [35]. Chestnut-backed antbirds are thought to be genetically monogamous, with extra-pair fertilization likely rare based on 2-egg clutches and extended parental care in the species [35]. Males and females are similar in body size but can be distinguished by ventral plumage coloration (i.e. females brown, males black) [35].

Previous studies report that males and females both produce short whistle-like two to four note songs while foraging or in response to intruders [35–37]. Males have been observed to sing more often than females and females reportedly sing at a slightly higher frequency (Hz), with the females often following their mate’s song to create a simple “duet” [35–37]. The birds have also been observed to sing “contagiously,” where the song of one bird elicits song from one or more neighbors, forming a chorus [35].

The breeding season of chestnut-backed antbirds is thought to extend from March to October [38]. Anecdotally, males have been reported to respond aggressively to playback year-round whereas females respond more in the beginning of the main breeding season [35]. This study was conducted from 26 July to 12 Aug 2016 on Barro Colorado Island (BCI), Panama (9°09’N, 79°51’W) and thus past the middle of the breeding season.

### Playback stimuli

**Fig 1:**
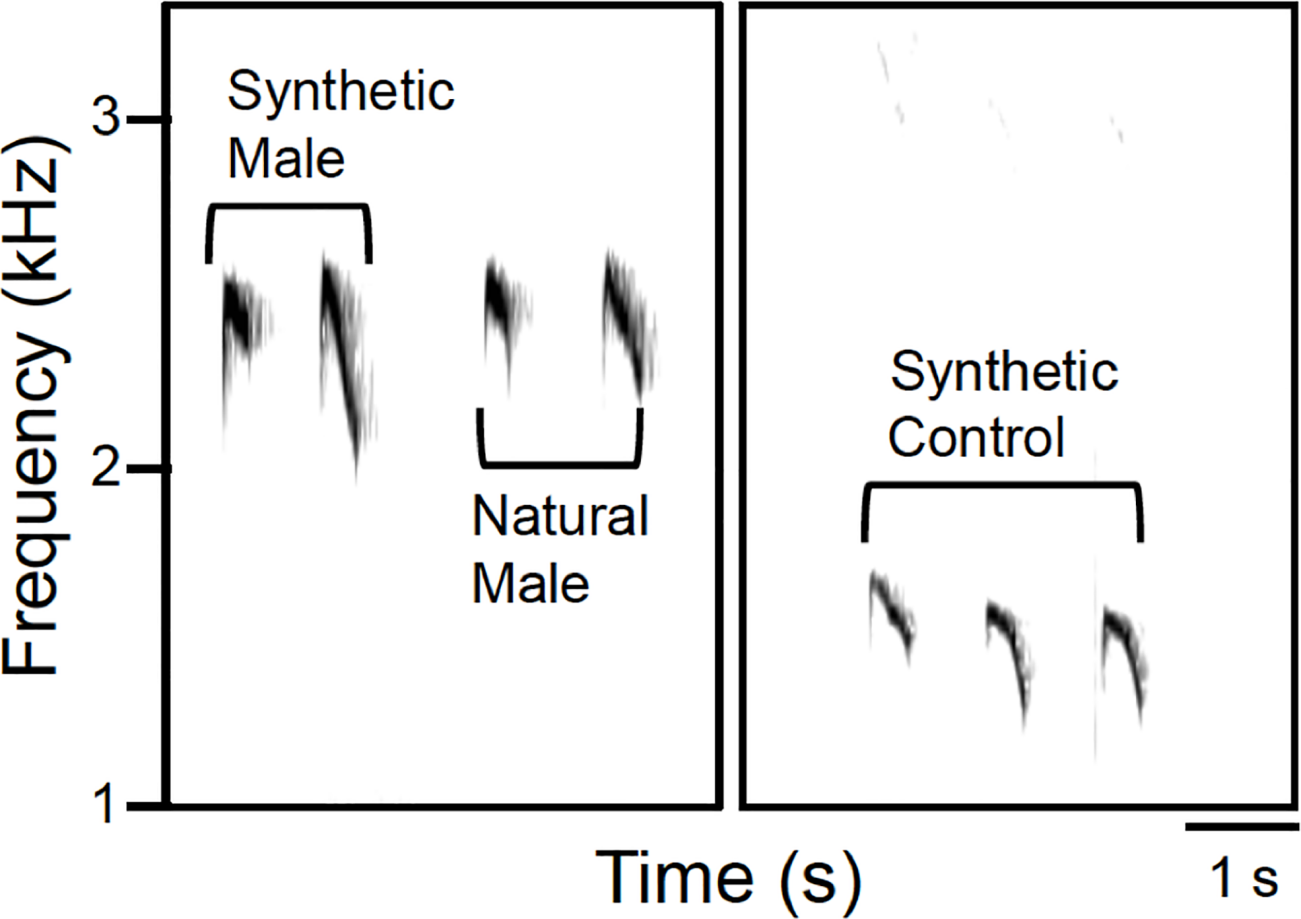
Playback stimuli. Spectrograms of natural two-note male chestnut-backed antbird (*Myrmeciza exsul)* song, synthesized version of the song, and synthesized song of black-throated trogon *(Trogon rufus)* used as a control. Synthesized songs were created based on the timing, amplitude envelope, and frequency characteristics of natural songs.

To test whether differences in song frequency (Hz) alone can trigger sex-specific vocal responses in this species, we used synthetic songs instead of natural songs, allowing us to manipulate only frequency. We created four types of synthetic songs for playback: male solo, female solo, male led duet, and a control stimulus (black-throated trogon song) using a custom MATLAB program (created by Edward W. Smith, S1 Code). One male song (two notes) was chosen from recordings made in Aug. 2014 on Barro Colorado Island as a model for the synthetic song [33] (Fig 1). We extracted timing, amplitude envelope, and frequency characteristics from the song using Raven Pro 1.4 (Bioacoustics Research Program, 2011) and entered those values into the MATLAB program, creating a synthetic song that sounded like the natural one (see S1 Table for parameters of all stimuli). Based on frequency differences between male and female song measured in the Aug. 2014 recordings, we created a typical “female” song that was the same as our “male” song in all parameters except that it was raised 200 Hz in frequency. To mitigate potential pseudoreplication, three sets of “male” and “female” songs were used: frequency parameters in version 2 were 20 Hz lower than in version 1 and in version 3 were an additional 20 Hz lower than in version 2. Duet stimuli were created by combining a two-note male song and a two-note female song, with the male song played first, separated by an inter-song interval of 2 s, matching a typical example of a duet in the Aug. 2014 recordings. In addition, songs of resident and intruder males for use in playbacks at a microphone array (see below) were created based on recordings made during this study. We chose black-throated trogon *(Trogon rufus)* song as a control stimuli because the birds are common at the study site and have a similar, although lower frequency, song (Fig 1). We synthesized trogon songs in the same manner as the chestnut-backed antbird songs, creating a set of three stimuli shifted in frequency by steps of 20 Hz. All stimuli were broadcast with ten seconds of silence between songs (or between duets).

### Playback technique

We first identified a total of 17 spatially distinct playback sites based on hearing spontaneously vocalizing chestnut-backed antbirds near the trails. To avoid sampling the same territory multiple times, we chose sites that were at least 150 m apart on the trail (see S1 Fig, S2 Table), approximately larger than the size of the birds’ reported 1 ha territories on Barro Colorado Island [39]. As the birds have been reported to rarely leave their territories [39], we think it is unlikely that we ever observed the same bird in multiple territories, despite not being able to use color-banded birds. The sites we used were not mapped out territories, so we also could not control for whether we were closer to the edge or the center of a territory.

We then performed at least one playback trial using each of the four types of synthetic songs at all the 17 different sites, which we presume to be in or near the territories of 17 pairs of chestnut-backed antbirds, though we cannot be certain there were females at every site. We randomly selected site, treatment type (male, female, duet, or control), and one of the three variants of each treatment type to ensure that all song types were eventually presented at all sites and then conducted a single playback trial (e.g. broadcast one of the three variants of the male song stimuli) at a site in the morning. All 17 sites received all 4 playback types and we also did a 5^th^ playback at 6 sites (3 male solo, 1 female solo, 2 duet). We used each song type variant 2–10 times and never used the same stimulus multiple times at the same site. In total, we conducted 74 playback trials (17 control, 18 female, 19 duet and 20 male). We conducted all playback trials for the first phase of the study between 0700 and 0930 hours, as the birds have been observed to sing most in the morning [33, 35, 39]. In the second phase of the study, playbacks at microphone arrays (described below) were conducted in the morning and early evening.

Sounds were broadcast using smartphones connected wirelessly to a pair of portable loud speakers (HMDX Jam Plus) at an amplitude similar to natural songs. These speakers were placed on the ground approximately 5 m apart. During duet playbacks, the male song was broadcast from one speaker and the female song from the other. During solo playbacks, either male, female, or control songs were played from one speaker. We recorded from approximately 10 m away using ZOOM H2n Handy Recorders connected to Sennheiser ME 66 microphones (flat frequency response between 200 and 6000 Hz (±2 dB)). We recorded in 16 bits at 48 kHz for five minutes before, during, and after the playback. During each 15 min trial, we noted the arrival of any chestnut-backed antbirds and the sex of the observed birds, identified by ventral plumage coloration. Analysis of recordings made with a microphone array (see below) confirmed that synthetic song amplitude was comparable to the amplitude of natural song produced by a bird at a similar distance from the microphone.

### Vocal responses to playbacks

For each recording period (i.e. before, during and after a playback), we manually delimited the boundaries of each recorded song using Raven Pro 1.4 and measured the number of songs, the number of notes in each song, and the following acoustic features of the vocalizations: high frequency, low frequency, duration, and bandwidth (high—low frequency). We created spectrograms using an FFT of 2046 in a Hann window with 50% overlap resulting in a temporal resolution of 23.2 ms and a frequency resolution of 21.5 Hz. We measured all songs observable on the spectrograms for each five-minute recording period before, during, and after the playback. Based on field notes, we labeled each song as being produced by an identified male or female. If the sex of the bird was not visually identified, then the songs were labeled as unknown. When more than one bird was singing at a time, we used relative amplitude and frequency to distinguish songs from different individuals.

### Movement responses to playbacks

In the second phase of the study, we set up a microphone array to quantify male and female movements and used time delays to determine the positions of singing birds in response to playbacks at six of the 17 sites. Due to time constraints, we could not set-up the array at all 17 sites. Given that territory boundaries were unknown, the array recordings allowed us to capture movements of singing birds as they approached the speaker independently of territory boundaries. The array consisted of six Behringer ECM8000 ultra-linear condenser microphones (omnidirectional frequency response from 20–20,000 Hz (± 2 dB)) connected by 14.7 m cables to a Zoom H6 recorder, which recorded each channel in 16 bits at 48 kHz. The microphones were oriented vertically on stands with the capsule 50 cm from the ground. We placed the recorder in the center of the array and tried to position the microphones 14.7 m apart to form a hexagon (S2 Fig). We then measured each microphone’s location using a 50 m tape measure. We placed the loudspeakers inside the array, approximately 10 m away from observers or the individual operating the recorder. At each site we conducted two playbacks, one in the morning and one in the evening after 1700 h.

To determine locations of a singing bird, we used Audacity 1.3.10-beta to combine the six channels into a six-track recording, and then made a separate file for each song. We then used Raven Pro 1.4 to calculate all possible pairwise spectral cross-correlations for the last note in a song and determine the difference in arrival time of the sound to each microphone. We used a 512 FFT with 50% overlap in a Hann window (temporal resolution 5.8 ms, frequency resolution 86.1 Hz). Additionally, all audio files were filtered with a bandpass filter (2-3 kHz) to remove background noise. Bird position was estimated using the arrival time differences and microphone locations with SoundFinder [40]. We estimated the error in the system by measuring the location of the loudspeaker and then used the program to localize the source of the speaker sound using five different songs. We excluded missing data and data with high error values. We also excluded one case where the bird appeared to move an unrealistic distance, e.g. more than 20 m, between successive calls because observations of vocalizing birds revealed that they either perched or hopped while singing.

### Duet measures

We defined a duet bout as beginning when an identified bird responded (the follower) to an identified bird of the opposite sex (initiator) within 5 s and a duet bout as ending when both birds stopped singing for at least 10 s within a recording period or when the recording period ended [41]. We measured the duration of each duet bout, the number of male and female songs within each bout, and the response latency of the follower to the initiator (i.e. onset of the follower’s song minus onset of the initiator’s song), which is a common measure used for the temporal coordination of duets [14].

### Song overlap

We compared song overlap observed to that predicted by chance assuming independence. We calculated observed overlap by summing each instance of overlap identified from times extracted from Raven Pro 1.4 selection tables. Predicted overlap for bird and playback was calculated by multiplying the proportion of time each bird spent singing by the proportion of time the playback songs were broadcast. Predicted overlap for multiple birds was calculated by multiplying the proportion of time each bird spent singing. The identity of more than one bird in a recording was deduced from the dominant song frequency, which invariably differed between individuals singing at a site. We quantified degree of overlap between bird and playback and between birds by subtracting predicted overlap from observed overlap.

### Statistical analyses

To determine whether acoustic features of 2-note songs recorded from birds whose sex we visually identified differed in high frequency, duration, and bandwidth, we first calculated mean values for identified birds at each site and then compared values between the sexes using t-tests. We chose these three acoustic features because they are weakly correlated and, therefore, provide largely independent characteristics of these simple songs (S3 Table). To determine how birds of each sex responded vocally to the playback stimuli we conducted a mixed model analysis of variance (ANOVA) using songs recorded per second from identified birds (excluding array recordings) as the dependent variable, site as a random effect, and playback treatment type (control, male, female, or duet) and period (before, during or after the playback) as fixed effects. We also used a mixed model ANOVA to determine whether acoustic features of songs from birds of known sex changed in response to playbacks by including site as a random effect and playback type (control, male, female, or duet) as a fixed effect. We used Tukey's Honest Significant Difference (HSD) test with alpha of 0.05 as a post-hoc test to identify differences among treatment types in each analysis. We used non-parametric Wilcoxon rank sum tests to compare male and female production of 3-note songs and the proportion of trials in which males and females were sighted in response to each playback type. We used a chi-square goodness-of-fit test to compare differences in which sex initiated duet bouts. In figures and text, error is indicated as standard error of the mean. We used JMP 10.1.02 for all statistical analysis.

### Ethics statement

The work was conducted with permission of the Smithsonian Tropical Research Institute IACUC, protocol 2016-0725-2019. All observations were restricted to the Barro Colorado National Monument and we obtained all required permits from the STRI SPO (Smithsonian Tropical Research Institute Scientific Permit Office).

## Results

### Description of male and female song

In total, we recorded 2,359 songs from males, 325 songs from females and 6,787 songs from birds of unidentified sex. At sites where we recorded songs from identified birds, the average number of songs (± SE) produced in response to a conspecific playback was 32.9 ± 1.9 for males (N = 36) and 22.2 ± 2.7 for females (N = 11). We observed males at all 17 sites and females at three sites. Females were usually less conspicuous and only ever observed at a site after a male had already appeared. Two-note songs from identified males and females differed significantly in high frequency (t = -4.4, P < 0.001; male = 2625.5 ± 15.2 Hz, female = 2773.1 ± 29.9 Hz), but not in duration (t = -0.51, P = 0.615; male = 1.168 ± 0.013 s, female = 1.183 ± 0.025 s) or bandwidth (t = -1.77, P = 0.085; male = 452.8 ± 12.9 Hz, female = 503.4 ± 25.4 Hz). Birds of unidentified sex produced songs that were, on average, in between the high frequencies of male and female songs (2676.1 ± 1.4 Hz). The mean high frequency of an unidentified bird was significantly higher than the high frequency of the identified male on one or more days at seven sites, consistent with the presence of cryptic females at those sites. Given that many of the other unidentified songs were lower in amplitude and frequency, we suspect they were made by male birds in neighboring territories.

### Description of duets

**Fig 2:**
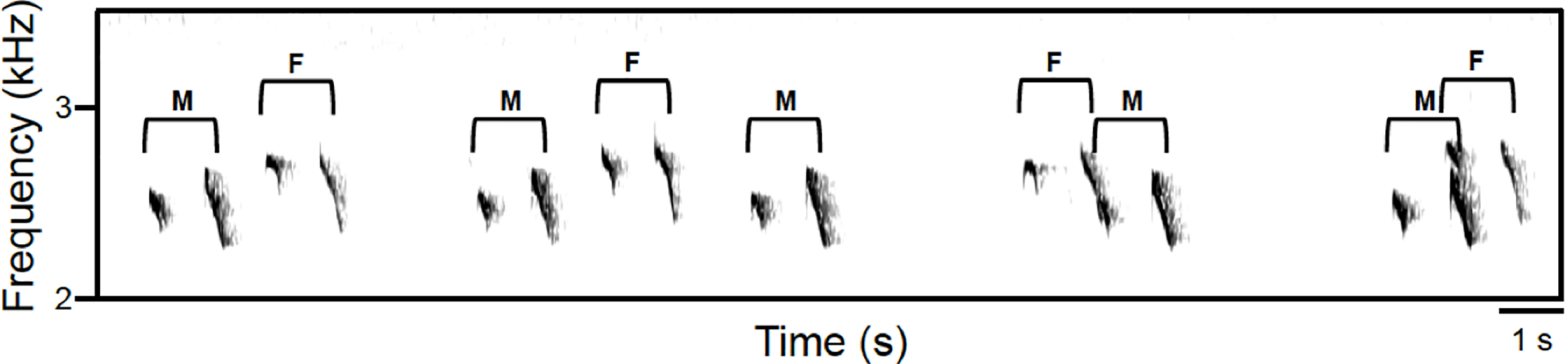
Duet song. Example of chestnut-backed antbird duet recorded in this study at Barro Colorado Island, Panama. M = male song, F = female song. Male and female birds were visually identified based on ventral plumage coloration.

From the full set of playback recordings, we detected 20 total duet bouts in 10 of the playback trials conducted at three of the 17 playback sites (see Fig 2 for example of duet song). These duet bouts included 440 male songs, accounting for 18.7% of total male songs, and 307 female songs, accounting for 94.5% of total female songs. Males initiated 60% of recorded duet bouts, while females initiated 40%, which did not significantly differ (Χ^2^ (1, N = 20) = 1.6, p = 0.206). The average duration of duet bouts was 150.94 ± 24.09 s. The average response latency of follower’s songs to initiator’s songs was 1.91 ± 0.18 s.

### Sex-specific responses to playbacks

**Fig 3:**
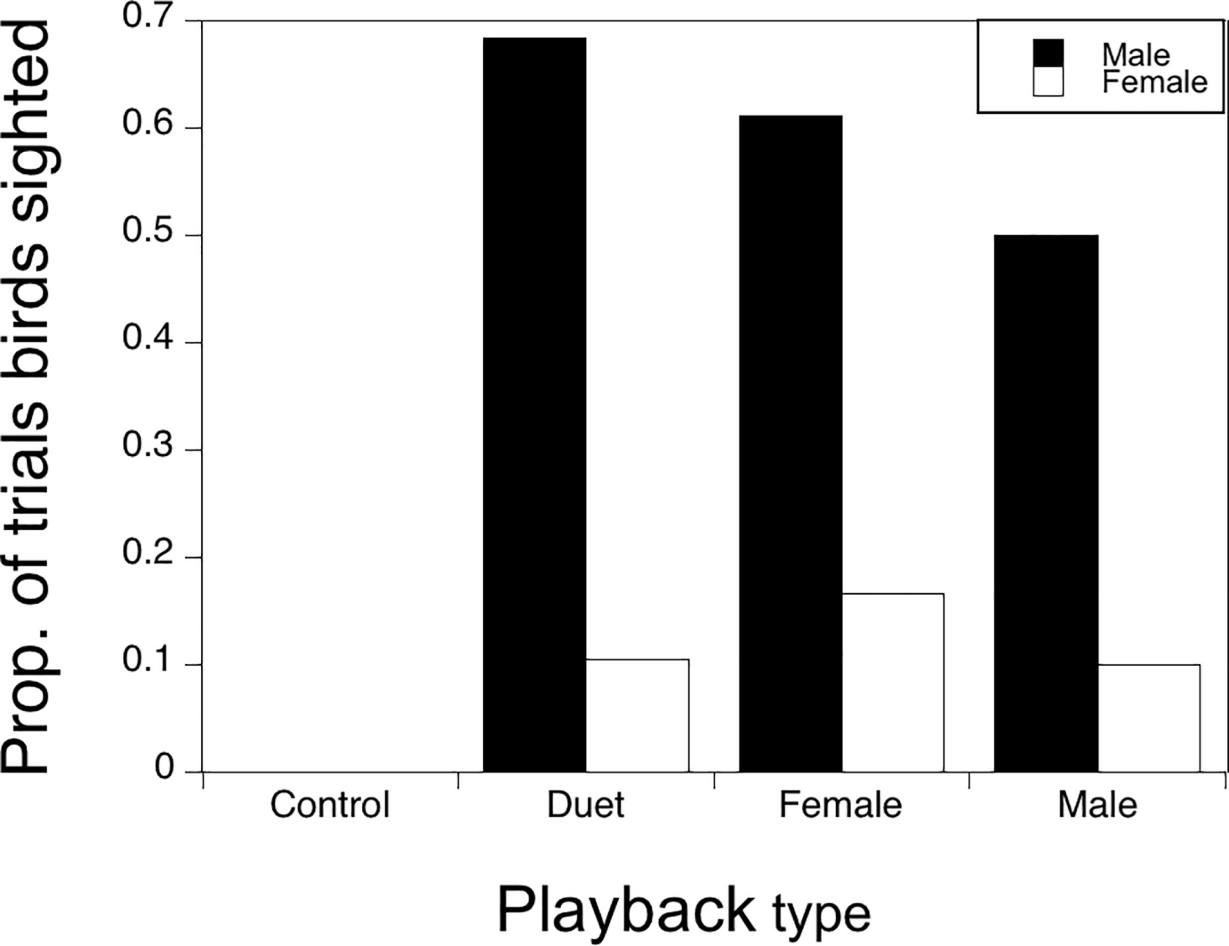
Sex differences in sighted birds. Proportion of trials in which a male or female chestnut-backed antbird was sighted in response to each playback type. Males were observed more often than females (58% vs 15% of playback trials, W = 217.5, P < 0.001; Wilcoxon rank sum test) in response to conspecific (non-trogon) playbacks. Females were also only ever observed at a site after a male had already appeared.

**Fig 4:**
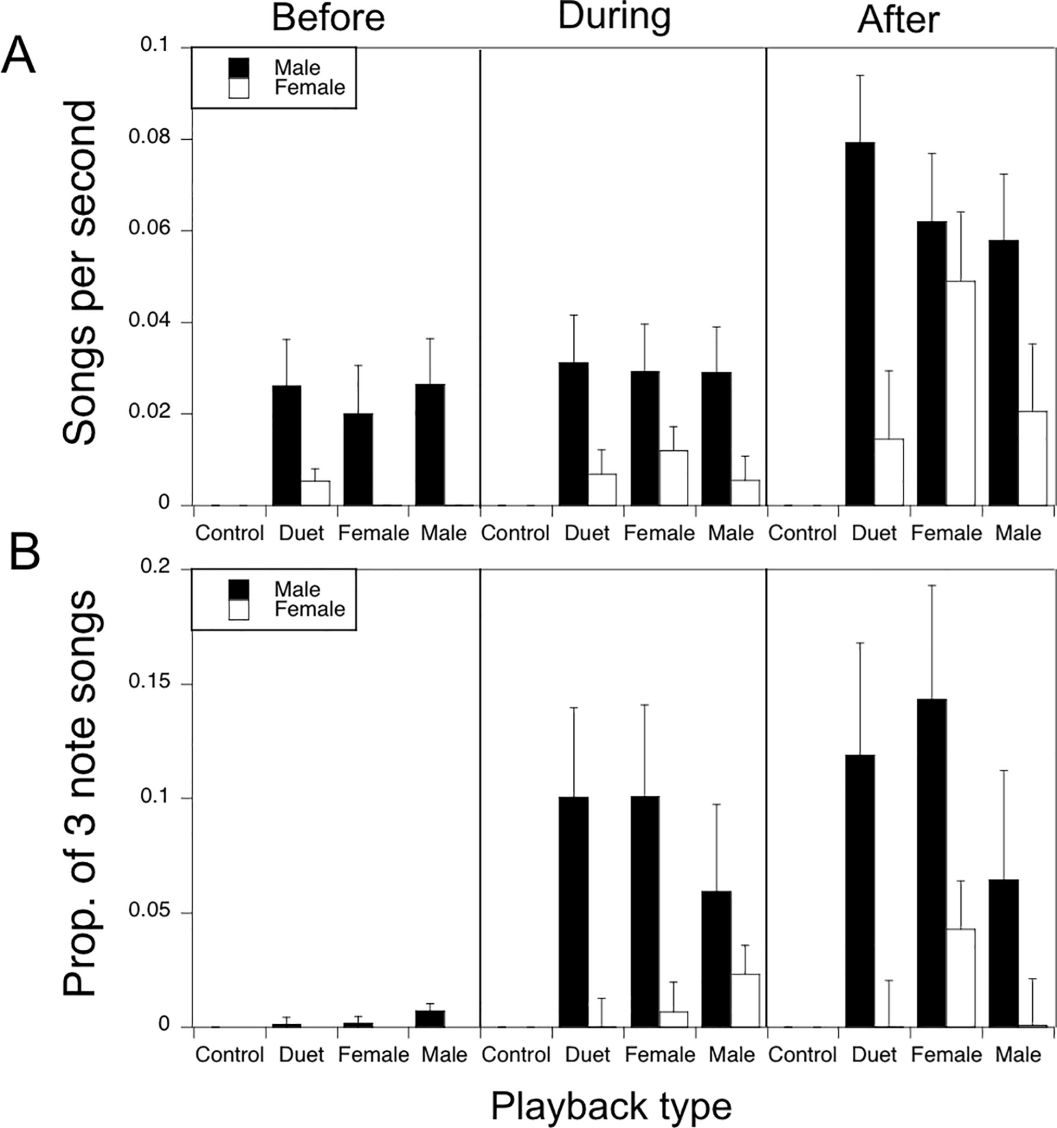
Sex differences in vocal response to playback. a) Song rate (±SE) in response to playback type for 5 min recordings before, during, and after playback. Mixed model analysis revealed that song rate depended on playback type and period for both males (playback type: F_3,216_ = 10.63, P < 0.001; period: F_2,216_ = 10.70, P < 0.001) and females (playback type: F_3,216_ = 2.77, P = 0.043; period: F_2,216_ = 5.79, P = 0.004). (b) Proportion of 3 note songs out of total songs (±SE) in response to playback type for 5 min recordings before, during, and after playback. Males produced a three-note song more often than females (W = 3068.5, P < 0.001; Wilcoxon rank sum test).

Overall, we observed males more often than females (58% vs 15% of playback trials, W = 217.5, P < 0.001; Wilcoxon rank sum test) in response to conspecific (non-trogon) playbacks (Fig 3). In addition, males responded with a greater song rate than females during playback trial periods where both a male and female were observed at a site (t = 2.96, df = 16, P < 0.009; two-tailed paired t-test). Mixed model analysis revealed that male song rate depended on playback (Fig 4A; F_3,216_ = 10.63, P < 0.001) and period (F_2,216_ = 10.70, P < 0.001) with no significant interaction. Site explained 23.3% of the variation in male song rate. Post-hoc analysis revealed no difference in male song rate in response to the three conspecific song stimuli but a significant difference to the trogon control stimulus, which was essentially ignored. Post-hoc analysis also showed that song rate after the playback was significantly higher than either during or before the playback. Female song rate also differed between playback types (F_3,216_ = 2.77, P = 0.043) and across the three playback periods (F_2,216_ = 5.79, P = 0.004) with the greatest response occurring after playback of a female song (F_1,216_ = 6.11, P = 0.014, post-hoc contrast). Site explained 21.2% of the variation in female song rate. Males produced a three-note song more often than females (Fig 4B; W = 3068.5, P < 0.001; Wilcoxon rank sum test).

To examine the birds’ movement behavior, we analyzed array recordings from seven playback trial periods in which birds vocalized at four of the six sites (we did not analyze recordings from the other two sites because birds did not vocalize). Using individually distinctive acoustic features we measured the time delays to all microphones and used them to reconstruct the movements of visually identified males and females. Overall, the minimum approach distance to the speaker was less for males (3.8 ± 1.0 m) than for females (5.8 ± 0.9 m). Males also began singing (after onset of recording) well before females (t = 2.64, P = 0.032; males = 391 ± 101 s, females = 673 ± 35 s). Consequently, the range of distances away from the speaker in which birds sang was greater for males (26.1 ± 5.9 m) than for females (14.2 ± 4.2 m).

### Changes in acoustic features based on playback treatment

**Fig 5:**
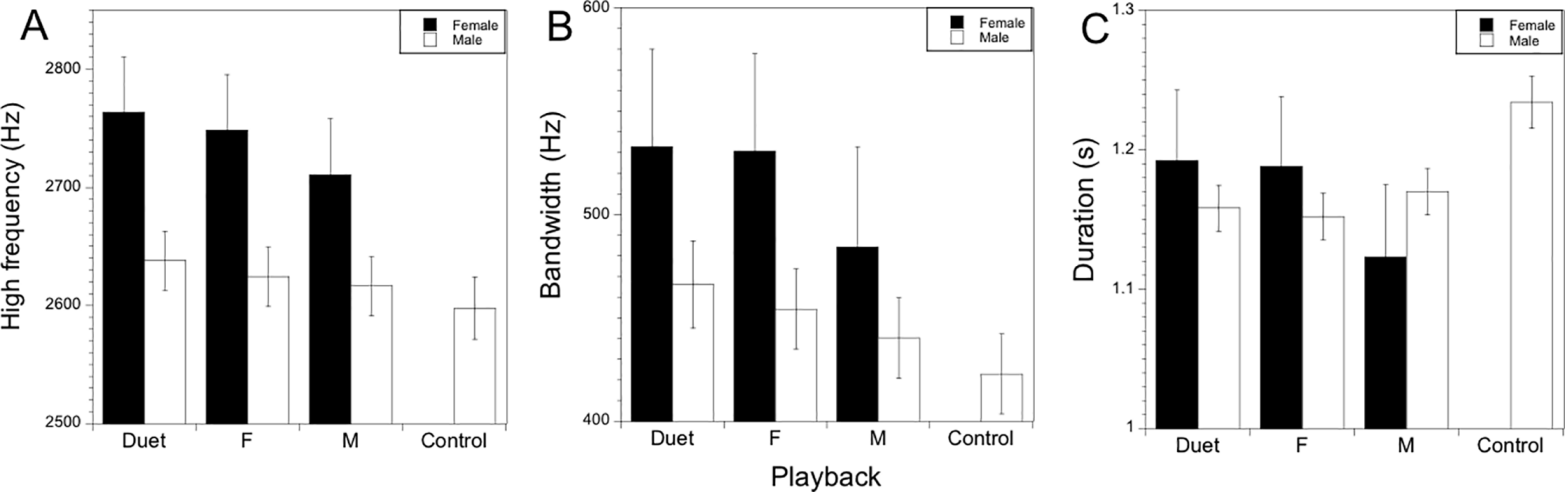
Changes in acoustic features in response to playback type. (a) High frequency for males and females in response to playback type. (b) Bandwidth for males and females in response to playback type. (c) Duration for males and females in response to playback type. Post-hoc comparisons indicated by capital letters for females and by lower case letters for males. If two types share the same letter, they are not different.

Using mixed models in which site was a random effect and treatment was a fixed effect, we found that high frequency, bandwidth, and duration changed between treatments for both males and females (Table 1; Fig 5). In all analyses, site explained a large fraction of the variation in the acoustic features. For example, site explained more than 80% of the variation in high frequency in both males and females. As the sites in general were similar in landscape and physical features, this result is consistent with individual differences among birds that are consistently at the same site. Supporting this interpretation, at one site one male consistently produced a 4-note song, which was an extremely rare song type across the population. That song was recorded in response to every type of conspecific playback presented at that site on different days, which is strong evidence that the same individual was present each time.

**Table 1:** Changes in acoustic features in response to playback type.

Sex	Variable	df	F	P	%var by site
**Female**	Max freq	2,310	27.4	< 0.001	81.6
	Bandwidth	2,310	9.5	< 0.001	63.9
	Duration	2,310	5.9	< 0.001	34.9
**Male**	Max freq	3,2040	32.2	< 0.001	87.1
	Bandwidth	3,2040	52.2	< 0.001	64.3
	Duration	3,2040	78.5	< 0.001	57.5

Results from six mixed model analyses with site as a random effect and treatment as a fixed effect show that three distinct acoustic features in both male and female songs differ in response to different types of playbacks.

### Song overlap

**Fig 6:**
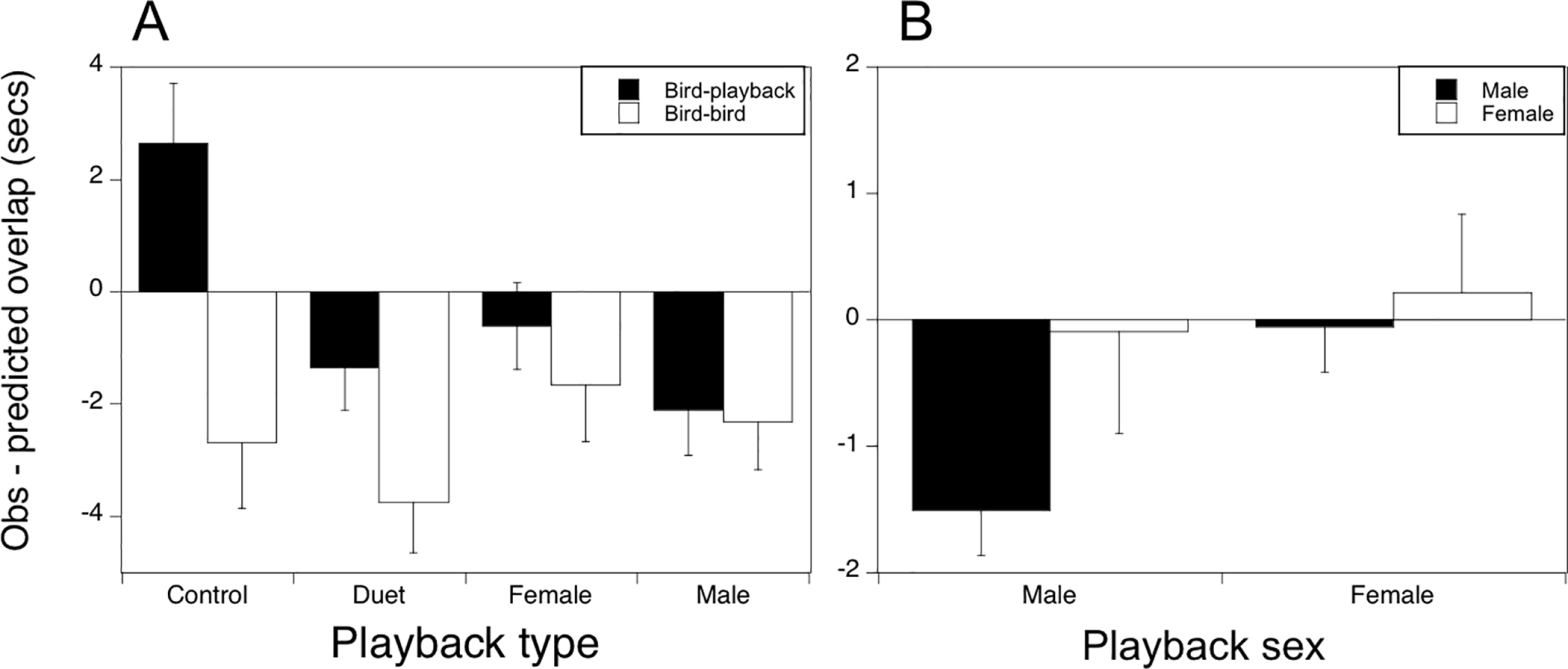
Song overlap with playback and other birds. (a) Song overlap in response to playback type for birds overlapping with the playback and birds overlapping with each other. Song overlap is the predicted overlap subtracted from observed overlap (±SE). For overlap of natural songs with the playback sounds, the difference between observed and predicted overlap depended on playback type (F_3,38_ = 3.78, P = 0.018). The birds avoided overlap with other conspecifics (not the playback) in all conditions and the duration of song overlap did not significantly differ based on playback treatment type **(**F_3,73_ = 1.34, P = 0.272) (b) Song overlap (±SE) by males and females with the “male” and “female” playback song. Observed male birds avoided overlap with male playback songs more than female playback songs (F_1,34_ = 8.13, P = 0.007).

For overlap of natural songs with the playback sounds, we found that the difference between observed and predicted overlap depended on playback type (F_3,38_ = 3.78, P = 0.018). Post-hoc analysis revealed less overlap than predicted in the duet and male solo conditions compared to controls (Fig 6A). For observed males and females, overlap with playback depended on playback type (F_3,34_ = 3.58, P = 0.024) and a post-hoc contrast revealed that observed male birds avoided overlap with male playback songs more than female playback songs (F_1,34_ = 8.13, P = 0.007) (Fig 6B). Also, we found that the birds avoided overlap with other conspecifics (not the playback) in all conditions and the duration of song overlap with conspecifics did not significantly differ based on playback treatment type (F_3,73_ = 1.34, P = 0.272). We did not observe enough cases of female song overlapping with the playback or conspecifics to determine if female song overlap differed from male song overlap.

## Discussion

In this study, we found that synthetic song playback differing in frequency triggered sex-specific responses in chestnut-backed antbirds. The sex differences observed are consistent with anecdotal accounts of vocal responses to playback of natural songs in this species [35, 39], indicating that the birds responded to the synthetic playback as if it was natural song. We also found evidence that the birds’ responses were dynamic rather than static, in that the birds changed the high frequency, bandwidth, and duration of their songs depending on the playback treatment and that the birds actively alternated their songs.

### Functions of duetting in chestnut-backed antbirds

We show that male chestnut-backed antbirds consistently responded to playbacks and often participated in defense alone. In cases where females approached the speaker, they always did so after the males, arguing against a joint-territory defense interpretation [13]. Results from the array recordings also show that males and females did not typically respond vocally to the playback in a joint display. Since males were very responsive to all conspecific playback treatments, males seem to be defending their territories from all conspecific intruders. Typically, we observed that males approached the speaker silently and then increased song rate after the playback ceased, perhaps assessing the intruder before responding. Our analysis of recorded duets in this species supports the interpretation that the birds did not coordinate song for joint-territory defense. Duets with response latencies less than 200 ms have been categorized as high temporal precision and those with greater response latencies as low temporal precision [20]. In chestnut-backed antbirds, duet response latencies were greater than 200 ms, indicating low temporal precision and a lack of evidence for joint territory defense [42].

Female chestnut-backed antbirds responded most strongly to female solo playback, suggesting their participation in duetting in this context is consistent with a mate guarding function [13]. In mate guarding, the guarding sex is also predicted to create the majority of duets by responding to its partner [20]. Here, females created duets by responding to male solo songs the majority (60%) of the time, consistent with a mate guarding function for female participation. But given that males created duets in 40% of observed cases, mate guarding may not be the only function of duets in this species. As the number of females recorded in this study was small, these results must be viewed as preliminary.

Another possibility is that chestnut-backed antbird duetting behavior may vary across the breeding season, as is the case for a number of species (e.g. rufous-and-white wrens (*Thryothorus rufalbus)*, [43]; Venezuelan troupial (*Icterus icterus*), [44, 45]; rufous hornero (*Furnarius rufus*), [46]), but not others (e.g. magpie-larks (*Grallina cyanoleuca*), [47]). For instance, in rufous-and-white wrens, females sing more often early in the year and their song output decreases as the breeding season progresses, reflecting a shift in the function of duets across the breeding season [43]. For chestnut-backed antbirds, Stutchbury et al [39] found that during the nonbreeding season (February), at least, females only occasionally sang and when they did, it was immediately after their mate’s song, consistent with our findings. Variation in response could also be due to the nesting cycle, i.e. females may be more responsive during periods when males are providing care, or due to the location of playback intrusion within the territory, i.e. a female may be more responsive to playback at the center of its territory rather than at the edges. Future studies are needed to determine the influence of these factors on the functions of duetting in this species.

In comparison to other neotropical suboscine passerines, vocal behavior in chestnut-backed antbird females is most similar to the warbling antbird (*Hypocnemis sp*.), where females in a playback experiment responded to same-sex solos more strongly than duets [19]. But, in that species both sexes seem to duet for mate guarding purposes. In the barred antshrike (*Thamnophilus doliatus*), on the other hand, females physically responded more intensely to female solos than duet playbacks, providing evidence for a form of mate guarding. But in terms of vocal behavior, both male and female barred antshrikes sang vigorously in response to all conspecific playback, arguing primarily for a joint territory defense function for duetting [48, 49]. Thus, compared to other suboscines in the region that have been studied, chestnut-backed antbirds appear unique in the extent of sex differences in duetting behavior. Yet those studies on other suboscines were conducted earlier in the breeding season than ours, so it is indeed possible that sex differences in the duetting behavior of chestnut-backed antbirds are less defined at that time period.

### Active avoidance of song overlap

Our results show that chestnut-backed antbirds actively avoid overlap with other individuals and that the intrusion of conspecifics does not affect the temporal synchrony of vocalizing pairs who are defending their territories. Specifically, we also found that male chestnut-backed antbirds modulate the timing of their songs based on the sex of intruders. As chestnut-backed antbirds maintain very stable territories and have been observed to sing “contagiously,” where the song of one bird elicits song from one or more neighbors [35], a possibility is that the birds avoid overlap in order to communicate their position to neighboring and intruding birds. Since chestnut-backed antbirds are abundant on Barro Colorado Island, high population density may provide selective pressure to avoid overlap and maximize information transfer [50, 51].

Though the birds overlapped songs less than predicted by chance, it is also possible that when overlap occurs, it is not accidental but rather serves as an intentional signal [52]. Studies have argued that song overlap functions, for instance, as an aggressive territorial signal [53], as a defensive withdrawal signal [54], or as a jamming signal of mates in response to unpaired sexual rivals [31]. Future experiments using overlapping duet song as a playback treatment and interactive playback where the broadcast song overlaps with singing birds would help to determine if song overlap has a signal function in this species.

### Changes in acoustic features of songs in response to playback

Because suboscines are thought to have limited ability to adjust the acoustic features of their songs, we predicted that spectral features of chestnut-backed antbird songs would remain static across playback treatments. Instead, we found that the birds varied the high frequency, bandwidth, and duration of their songs depending on the type of playback treatment, suggesting a communicative function independent of noise avoidance–although it is possible that the low frequency of our control song reduced the need to modify frequency. Rather than noise avoidance, our results are more similar to a study on ocellated antbirds *(Phaenostictus mcleannani)* showing that males increase the frequency of their songs in aggressive interactions [55]. A possibility is that the chestnut-backed antbirds were engaging in frequency matching as occurs in some songbird species (e.g. [56]). But in our case, both male and female chestnut-backed antbirds responded with the highest frequencies during duet playbacks, providing evidence against a simple frequency matching interpretation. As we did not use banded birds or known territories, we cannot definitively rule out the possibility that the same bird was sampled at more than one site, despite the highly sedentary nature of the species [39], but the strong effect of site on acoustic features and the array results showing that birds do not sing until they are 25 m away from the speaker are consistent with a single pair at each site.

## Conclusions

Using synthetic song playback, we showed that changes in song frequency (Hz) are sufficient to trigger sex-specific responses in a suboscine passerine, the chestnut-backed antbird. These sex-specific responses suggest that males sing in response to intruders for general territory defense and females join in for mate guarding. This means that males and females perceive the frequency of conspecific song and integrate that information to produce sex-specific responses, including adjustments in the timing and frequency of their own vocalizations. As the scope of this study was limited, future studies are necessary to determine how and why these suboscines modulate the acoustic features of their songs and to determine whether the functions of duetting vary across behavioral context and season for this species. But, despite their simple songs, these suboscine passerines are engaging in a complex system of communication and dynamic form of sensorimotor integration.

## Supporting information

S1 Code Implementation of synthetic song using MATLAB. (RTF)

S1 Fig Map of all playback sites on Barro Colorado Island, Panama. Labels denote the name of the trails. FC = Fairchild, F = Fausto, L = Lathrop, D = Donato, B = Barbour, W = Wheeler, SM = Snyder Molino. Coordinates for each playback site are available in S2 Table.
(PDF)

S2 Fig Example set up of six-microphone array for sound localization. (PDF)

S1 Table Parameters for synthesized stimuli. (XLSX)

S2 Table Coordinates for each playback site. (PDF)

S3 Table Correlations between high frequency, duration, and bandwidth. Correlations for high frequency, duration, and bandwidth using mean values from the 2^nd^ note of songs from visually identified male and female birds. These acoustic features are weakly correlated and, therefore, provide largely independent characteristics of the songs.
(PDF)
