# Supplementary material for: Dynamic sex-specific responses to synthetic songs in a duetting suboscine passerine
Source: PLoS One. 2018 Aug 29;13(8):e0202353. doi: 10.1371/journal.pone.0202353 (PMC6114868; doi:10.1371/journal.pone.0202353)
Supplement: S1 Code — (RTF) [file pone.0202353.s001.rtf]

function syllable = make_3Part_syllable_sloped ...     ( T0, T1, T2, T3, ...    A0, A1, A2, A3, ...    F0, F1, F2, F3, Harm_levels_dB, ...    slopes, shapes, white_noise_level, fs, ...    DO_PLOT_EACH_HARMONIC_COMPONENT )%      Ed Smith,   May 2016%      T0 is starting time of first segment, T1 is end time of first segment and start of second, T2 is end of second segment and start of third, T3 is end of third segment. A0 is amplitude level for T0, A1 for T1, and so on. F0 is frequency for T0, F1 for F1, and so on. Slopes can be ‘quadratic’ or ‘linear.’ If ‘quadratic’, shapes can be ‘convex’ or ‘concave’ % time axes for each segmentdt = 1 / fs;dur_01 = T1 - T0;dur_12 = T2 - T1;dur_23 = T3 - T2;t_01 = 0 : dt : (dur_01-dt);t_12 = 0 : dt : (dur_12-dt);t_23 = 0 : dt : (dur_23-dt); % compute points in each segmentnum_points_01 = numel ( t_01 );num_points_12 = numel ( t_12 );num_points_23 = numel ( t_23 );N = num_points_01 + num_points_12 + num_points_23 ; % determine Amplitude Envelope A_vec ( t )A_01 = linspace ( A0, A1, num_points_01 );A_12 = linspace ( A1, A2, num_points_12 );A_23 = linspace ( A2, A3, num_points_23 );A_vec = [ A_01, A_12, A_23 ]; N = N - 2; % Since two points will be dropped during concatenationA_vec = A_vec(1:(end-2)); % time axis for the complete syllablet = ( 0:(N-1) ) * dt; syllable = zeros ( 1, N );num_harms = numel ( Harm_levels_dB );for harmonic_index = 1 : num_harms        f0 = harmonic_index * F0;    f1 = harmonic_index * F1;    f2 = harmonic_index * F2;    f3 = harmonic_index * F3;        % Make first segment:    if isempty ( shapes(1) )        seg_01 = chirp( t_01, f0, t_01(end), f1, char(slopes(1)) );    else        seg_01 = chirp( t_01, f0, t_01(end), f1, char(slopes(1)), 0, char(shapes(1)) );    end;    end_pha = my_rad2deg(acos(seg_01(end)));    % See whether seg_01 ends in either QII or QIV and adjust end_phase as needed:    slope_at_end_of_segment = ( seg_01(end) - seg_01(end-1) ) / dt;    if (end_pha < (pi/2)) && (slope_at_end_of_segment < 0) % Segment end in quadrant II?        end_pha = pi - end_pha; % reflect end_pha about X = pi    end;    if (end_pha > pi) && (slope_at_end_of_segment > 0) % Segment end in quadrant IV?        add_on = 2 * ( (pi*3/2) - end_pha );        end_pha = end_pha + add_on; % reflect end_pha about X = pi    end;        % Make second segment:    if isempty ( shapes(2) )        seg_12 = chirp( t_12, f1, t_12(end), f2, char(slopes(2)), end_pha );    else        seg_12 = chirp( t_12, f1, t_12(end), f2, char(slopes(2)), end_pha, char(shapes(2)) );    end;    end_pha = my_rad2deg(acos(seg_12(end)));    % See whether seg_01 ends in either QII or QIV and adjust end_phase as needed:    slope_at_end_of_segment = ( seg_12(end) - seg_12(end-1) ) / dt;    if (end_pha < (pi/2)) && (slope_at_end_of_segment < 0) % Segment end in quadrant II?        end_pha = pi - end_pha; % reflect end_pha about X = pi    end;    if (end_pha > pi) && (slope_at_end_of_segment > 0) % Segment end in quadrant IV?        add_on = 2 * ( (pi*3/2) - end_pha );        end_pha = end_pha + add_on; % reflect end_pha about X = pi    end;        % Make third segment:    % Note that we do not care about the ending phase of the last segment.    if isempty ( shapes(3) )        seg_23 = chirp( t_23, f2, t_23(end), f3, char(slopes(3)), end_pha );    else        seg_23 = chirp( t_23, f2, t_23(end), f3, char(slopes(3)), end_pha, char(shapes(3)) );    end;        Harm_level_linear = 10 .^ (Harm_levels_dB(harmonic_index)/20);    signal_i = Harm_level_linear * [ seg_01 seg_12(2:end) seg_23(2:end) ];        if DO_PLOT_EACH_HARMONIC_COMPONENT        figure;        plot ( t, signal_i );        title ( [ 'Harmonic Component number ' num2str(harmonic_index) ] );    end;        syllable = syllable + signal_i ;    end; syllable = syllable .* A_vec;BG_noise = white_noise_level * randn ( 1, N );syllable = syllable + BG_noise; return;
