# Supplementary figures and images for: Dynamic sex-specific responses to synthetic songs in a duetting suboscine passerine
Source: PLoS One. 2018 Aug 29;13(8):e0202353. doi: 10.1371/journal.pone.0202353 (PMC6114868; doi:10.1371/journal.pone.0202353)

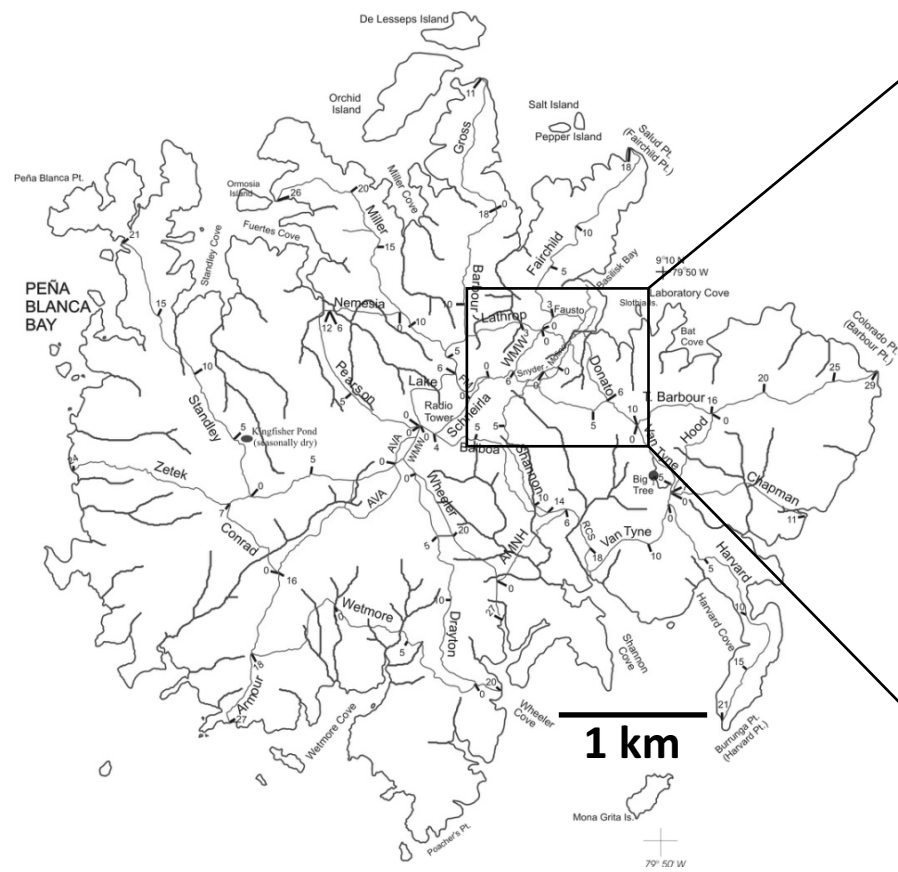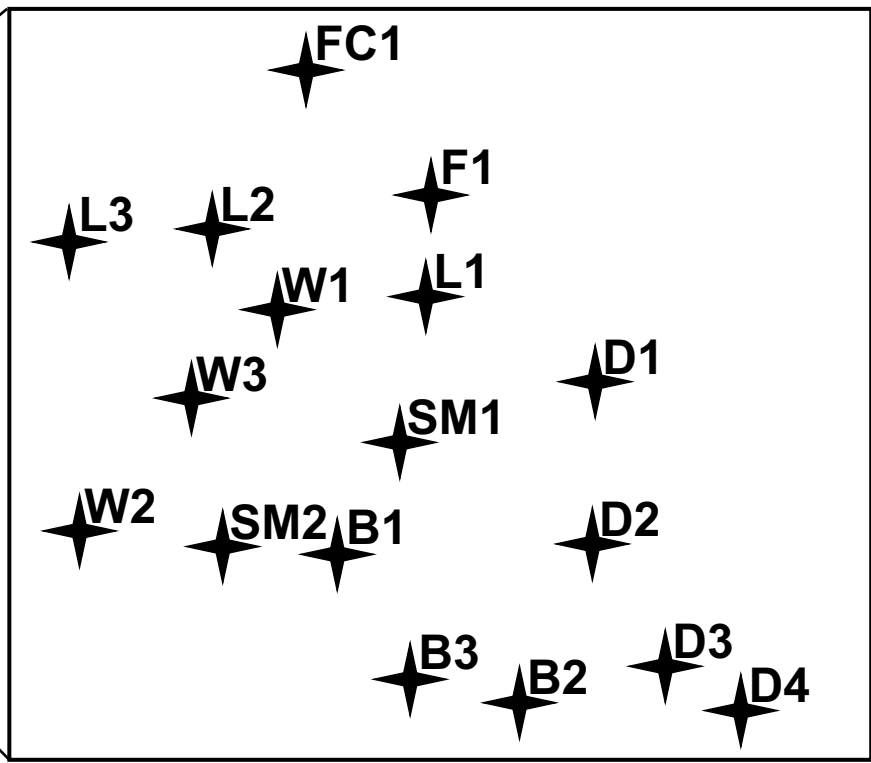

Supplement: S1 Fig — Labels denote the name of the trails. FC = Fairchild, F = Fausto, L = Lathrop, D = Donato, B = Barbour, W = Wheeler, SM = Snyder Molino. Coordinates for each playback site are available in S2 Table. (PDF) [file pone.0202353.s002.pdf]

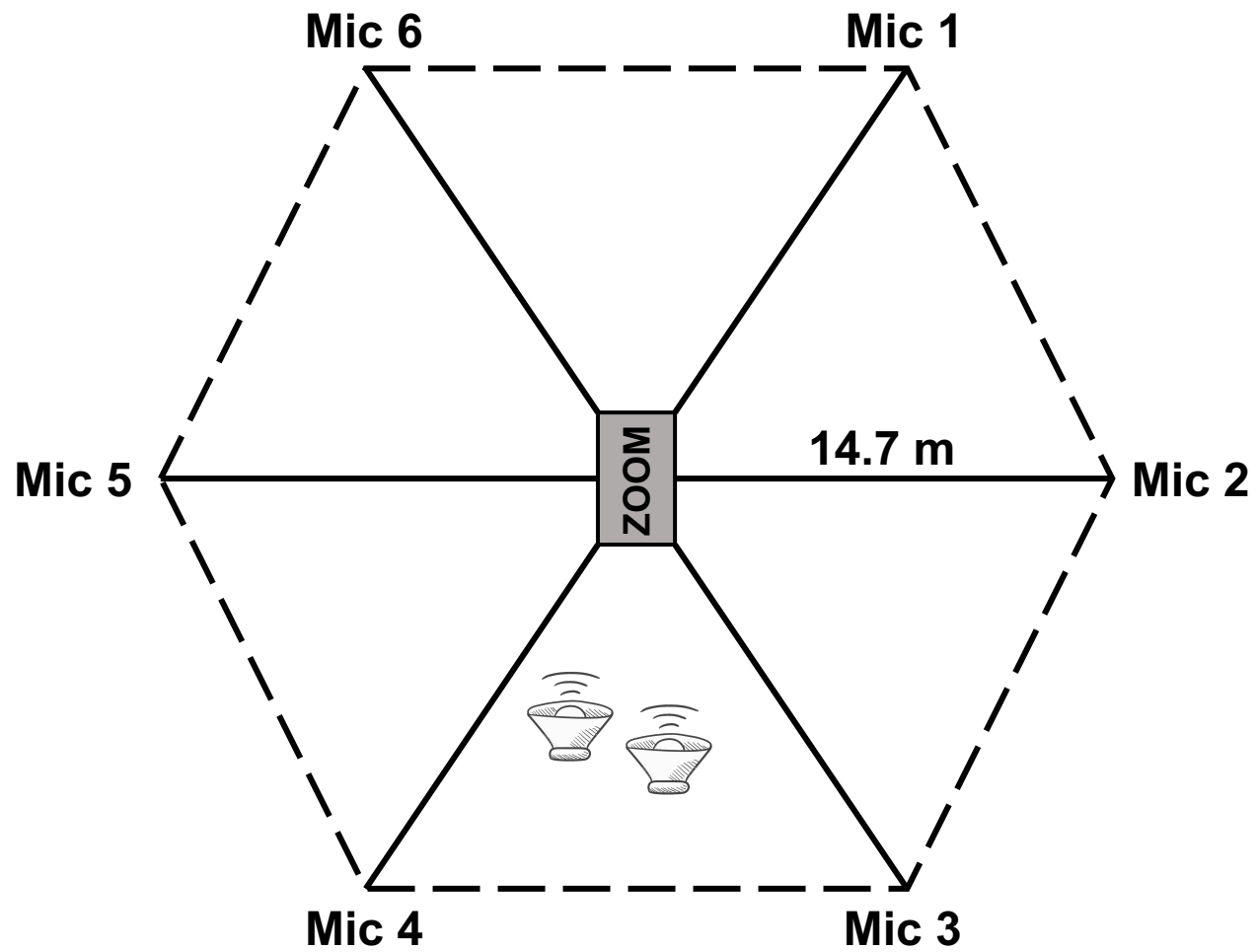

Supplement: S2 Fig — (PDF) [file pone.0202353.s003.pdf]
