# Supplementary material for: Dynamic sex-specific responses to synthetic songs in a duetting suboscine passerine
Source: PLoS One. 2018 Aug 29;13(8):e0202353. doi: 10.1371/journal.pone.0202353 (PMC6114868; doi:10.1371/journal.pone.0202353)
Supplement: S2 Table — (PDF) [file pone.0202353.s005.pdf]

Site	Latitude	Longitude
Barbour 1	9.160790°	-79.839710°
Barbour 2	9.159140°	-79.837590°
Barbour 3	9.159440°	-79.838784°
Donato 1	9.162420°	-79.836810°
Donato 2	9.160860°	-79.836740°
Donato 3	9.159560°	-79.836010°
Donato 4	9.159022°	-79.835237°
Fairchild 1	9.165994°	-79.839926°
Fausto 1	9.164972°	-79.838226°
Lathrop 1	9.163460°	-79.838600°
Lathrop 2	9.164320°	-79.840970°
Lathrop 3	9.164133°	-79.842477°
SnyderM 1	9.161940°	-79.838930°
SnyderM 2	9.160867°	-79.840858°
Wheeler 1	9.163470°	-79.840230°
Wheeler 2	9.161000°	-79.842450°
Wheeler 3	9.162459°	-79.841197°
