# Supplementary material for: Dynamic sex-specific responses to synthetic songs in a duetting suboscine passerine
Source: PLoS One. 2018 Aug 29;13(8):e0202353. doi: 10.1371/journal.pone.0202353 (PMC6114868; doi:10.1371/journal.pone.0202353)
Supplement: S3 Table — Correlations for high frequency, duration, and bandwidth using mean values from the 2nd note of songs from visually identified male and female birds. These acoustic features are weakly correlated and, therefore, provide largely independent characteristics of the songs. (PDF) [file pone.0202353.s006.pdf]

Variable	by Variable	Correlation	Lower 95%	Upper 95%	P-value
Mean(Bandwidth)	Mean(High Freq)	0.17	-0.154	0.461	0.301
Mean(Duration)	Mean(High Freq)	0.069	-0.252	0.376	0.677
Mean(Duration)	Mean(Bandwidth)	0.27	-0.05	0.539	0.097
